# Contributions of whole-genome sequencing to the epidemiological monitoring of *Campylobacter* spp. in France

**DOI:** 10.1128/aac.00193-26

**Published:** 2026-05-29

**Authors:** Quentin Jehanne, Johanna Aptel, Astrid Ducournau, Lucie Bénéjat, Moeava Martin, Christophe Rodriguez, Philippe Lehours

**Affiliations:** 1 National Reference Centre for Campylobacters & Helicobacters, Bordeaux, France; 2 Inserm, UMR 1312, BRIC, BoRdeaux Institute of onCology, University of Bordeaux, Bordeaux, France; 3 Plateforme GenoBioMICS, CHU Henri Mondor 378967, Créteil, Île-de-France, France; University of Pennsylvania Perelman School of Medicine, Philadelphia, Pennsylvania, USA

**Keywords:** *Campylobacter*, resistance, WGS

## Abstract

Next-generation sequencing techniques have revolutionized the epidemiology and understanding of *Campylobacter* spp. infections. In this study, we present the results of a next-generation sequencing (NGS) analysis conducted in 2024 on 2,360 *Campylobacte*r spp. strains isolated in France, including 1,959 *Campylobacter jejuni,* 358 *Campylobacter coli*, and 43 *Campylobacter fetus* strains. We describe the diversity of circulating strains and provide an overview of the main mechanisms of antibiotic resistance. We identified a variety of resistance mechanisms, particularly for resistance to group A penicillins. Notably, we identified a novel promoter upstream of the gene encoding a beta-lactamase. Using source attribution markers for *C. jejuni* and *C. coli,* we identified poultry as the main vector for infections by these two species. However, ruminant meat accounts for a significant proportion of *C. jejuni* infections in France. This study demonstrates how a sequencing strategy can generate large-scale and high-resolution data for understanding *Campylobacter* spp. infections.

## INTRODUCTION

The epidemiology of *Campylobacter* spp. infections in humans has recently been revolutionized by improved detection of campylobacteriosis by PCR, with greater sensitivity than the culture historically developed by Butzler and Skirrow in the 1970s. Owing to culturomics, the role of *Campylobacter* spp. in the epidemiology of acute infectious diarrhea is better understood (1). *Campylobacter* is now considered the main etiology, even more central than salmonellosis (2).

For more than 10 years, clinical laboratories in France have been equipped with PCR devices capable of diagnosing intestinal infections using multiplex panels that include *Campylobacter* species among the detection targets. These kits systematically detect *Campylobacter jejuni* and *Campylobacter coli*. Some kits also detect *Campylobacter upsaliensis* and *Campylobacter lari* (3–6). In many laboratories, selective agar is inoculated only with stool samples that tested positive for *Campylobacter* spp. by PCR, introducing a potential epidemiological bias regarding the diversity of species found in culture. In other laboratories, all *Campylobacter* spp. cultures have simply been discontinued, with the argument that macrolide resistance rates are low in France (<1% for *C. jejuni* and between 5% and 10% for *C. coli*) and that, if necessary, clinicians can safely prescribe antibiotic therapies like oral azithromycin. Nevertheless, *Campylobacter fetus* remains the third most commonly isolated species from stool samples in France each year. Some European and international microbiologists culture *Campylobacter* spp. from stool samples on agar plates incubated at 42°C (7). While this temperature is suitable for thermophilic species, such as *C. jejuni* and *C. coli*, it is too high for *C. fetus*. Therefore, this method introduces an epidemiological bias.

Nevertheless, *Campylobacter* culturomics still plays an important role (8). It is the only approach that can ensure the viability of bacteria, whereas PCR only detects the presence of DNA from the targeted species in the detection panel used (6). *Campylobacter* culture also allows the isolation of all potentially pathogenic species. Some studies have also shown that combining selective media with a cellulose nitrate membrane filtration technique allows the isolation of rarer Campylobacters, particularly those belonging to the so-called anaerobic *Campylobacter* group (e.g., *Campylobacter concisus*) (9). Owing to culture, new species are regularly described (e.g., *Campylobacter armoricus, Campylobacter ornithocola,* and *Campylobacter vicugnae*), demonstrating the broad diversity of this bacterial genus and the likely wide variety of contamination reservoirs for humans, comprising animals (10–13). To date, 52 validated species and 14 subspecies of *Campylobacter* have been described (https://lpsn.dsmz.de/search?word=campylobacter).

Another advantage of culture is that it allows the antibiotic susceptibility of the isolated strain to be determined. Resistance is carried primarily by the bacterial chromosome (resistance to group A penicillins, macrolides, and quinolones in particular) and sometimes on plasmids, particularly for aminoglycosides, tetracyclines, and macrolides (14–16).

In France, the National Reference Center for Campylobacters and Helicobacters (NRCCH) is responsible for monitoring *Campylobacter* spp. infections in humans. The NRCCH operates a surveillance network composed of private and hospital medical analysis laboratories. It receives approximately 5,000 strains of *Campylobacter* spp. each year, mainly *C. jejuni, C. coli*, and *C. fetus*. The strains are systematically identified by matrix-assisted laser desorption ionization–time of flight (MALDI-TOF) mass spectrometry (17). Until the end of 2023, all the strains were tested using the disc diffusion method. Each strain is accompanied by an information sheet requesting the patient data, the site of isolation (stool, blood culture, or other), the identified species, the nature of the case (isolated or grouped), the notion of travel, and the origin of the contamination. These data are rarely reported.

To better track the potential sources of contamination in France, the NRCCH implemented a sequencing strategy between 2017 and 2023. This addition allowed us to identify source attribution markers for *C. jejuni* and *C. coli* (18, 19). Campylobacteriosis in France due to *C. jejuni* originates from poultry and ruminant meat, whereas for *C. coli,* the poultry reservoir is largely predominant (20). The increase in next-generation sequencing (NGS) strategies within our laboratory has been accompanied by the implementation of a bioinformatics analysis pipeline. This pipeline allows us to identify our strains by genus and species, perform MLST typing, test our source attribution markers for *C. jejuni* and *C. coli*, and characterize the resistome. This strategy has made it possible in the past to characterize the resistance of Campylobacters in France, resistance to gentamicin, carbapenems, amoxicillin-clavulanic acid, and macrolides, notably with the emergence of erythromycin methyltransferases in France (14, 21–23).

At the beginning of 2024, we decided to routinely use this complete pipeline to provide our corresponding laboratories with results based on NGS analyses rather than traditional phenotypic results. We apply this strategy to all strains sent to us for which the laboratory provides us with the results of the antibiogram. These strains are subcultured upon receipt, identified by MALDI-TOF, and then sent to a sequencing platform. One week after shipment, the recovered sequences are automatically analyzed on the pipeline. Any discrepancy in identification is reported, and any discrepancy between the resistance phenotype entered and the resistome obtained is verified *in vitro*.

The purpose of this article was to summarize the results obtained in 2024 at the NRCCH using NGS for *C. jejuni, C. coli*, and *C. fetus*.

## MATERIALS AND METHODS

### Selection and isolation of clinical isolates

In 2024, the NRCCH received a total of 5,124 *Campylobacter* species isolates, mainly *C. jejuni*, *C. coli,* and *C. fetus*, accounting for approximately 60%, 10%, and 1% of the total data set, respectively. Isolates received with antibiogram data were routinely selected for whole-genome sequencing (WGS), which resulted in a subtotal of 2,360 clinical isolates of *C. jejuni* (*n* = 1,959), *C. coli* (*n* = 358), and *C. fetus* (*n* = 43). Each isolate was initially isolated on Columbia blood agar (CBA) plates supplemented with 5% sheep blood (Thermo Fisher Scientific, MA) and incubated at 37°C in a jar. An Anoxomat microprocessor (Mart Microbiology BV, Lichtenvoorde, the Netherlands) was used to create a microaerobic atmosphere of 79.7%N_2_, 7.1%CO_2_, 6.1%O_2_, and 7.1%H_2_.

### Bacterial identification and antibiotic susceptibility testing

Bacterial species were identified from pure cultures using MALDI-TOF mass spectrometry, as previously described (17). In cases of discrepancy between the submitting laboratory’s AST results and the NRCCH NGS results, additional antimicrobial susceptibility testing (AST) was performed using the Etest method to determine the minimum inhibitory concentrations (MICs) of erythromycin, ampicillin, amoxicillin–clavulanic acid, ciprofloxacin, gentamicin, and tetracycline. An inoculum at the 0.5 McFarland standard of pure *Campylobacter* was subcultured on Mueller–Hinton (MH) agar supplemented with 5% defibrinated horse blood (MH-F) and 20 mg/L nicotinamide adenine dinucleotide (β-NAD) (bioMérieux, Marcy l’Etoile, France), and the samples were incubated for 48 h in a microaerobic environment at 36°C. MICs were determined on MH-F using Etest strips (bioMérieux), read by two independent readers, and interpreted on the basis of the CASFM/EUCAST 2024 recommendations for *Campylobacter* species (https://www.sfm-microbiologie.org/wp-content/uploads/2024/06/CASFM2024_V1.0.pdf).

### Whole-genome sequencing and assembly of *Campylobacter* isolates

Whole-genome sequencing was performed on previously obtained pure subcultures of each isolate. The bacterial suspension was sent by regular mail to the Henri Mondor sequencing platform (Créteil, France). Paired-end sequencing was performed using an Illumina NovaSeq 6000. The raw sequencing data (.fastq) were cleaned using Sickle v1.33 (24), and the genomes were *de novo* assembled using SKESA v2.5.1 (25). On average, 94.28% ± 2.8% of the reference genome was covered, and the assembled genomes were 1,680,284 bp ± 155 kbp in size on average, with 36.36 ± 36.7 contigs and a GC percentage of 30.52% ± 1.1% (Table S1).

### Whole-genome analyses

Species were confirmed by the molecular average nucleotide identity (ANI) method using FastANI v1.33 (26): a threshold of ⩾95% was assigned to validate the species identification. Sequence types (STs), clonal complexes (CCs), and core-genome MLST were identified using the PubMLST *C. jejuni* and *C. coli* profile database and tools (cgMLST Campylobacter scheme v1.0). Clustering of *Campylobacter* isolates into clones was computed using the hierarchical clustering algorithm of ReporTree v2.6.0, with a maximum of 25 and 4 allelic differences (27), and distance trees were constructed using GrapeTree v1.5.0 (28). Antimicrobial resistance (AMR)-associated mechanisms were determined using the Blastn command line tool v2.15.0+ (29), combined with multiple genes, proteins, and mutation databases, namely the NCBI, CARD, and ResFinder databases, as well as the in-house NRCCH *Campylobacter* resistance database. Source attribution within the chicken, ruminant, and environmental reservoirs for *C. jejuni* and the chicken, ruminant, and pig reservoirs for *C. coli* was estimated using STRUCTURE (30), combined with host-segregating genes (18) and mutations (19). Finally, genome annotations were performed using Prokka v1.14.5 (31), and plasmid DNA was predicted using the RFPlasmid v1.0 tool (32).

## RESULTS

### Description of the origin of the strains tested in our study

In 2024, the NRCCH sequenced 2,360 genomes: 1,959 *C. jejuni*, 358 *C. coli*, and 43 *C. fetus* (complete data of the studied isolates are available in Table S1). The distributions by sex and age are shown in Table 1. *C. fetus* was isolated from older patients compared with those of *C. jejuni* and *C. coli*. Isolates were obtained from diverse metropolitan French regions and from overseas territories, as displayed in Fig. S1. *C. jejuni* and *C. coli* strains were isolated predominantly from stools (Table 2). Bacteremia predominantly involved *C. fetus*, which was the only species isolated from joint fluid or cerebrospinal fluid. Among the 2,360 strains included in our study, 1,240 were isolated cases of infection, 109 were grouped cases (mostly familial, *n* = 101/109, 92.7%), and 1,011 had no information available. Few identification discrepancies between the identification provided by the sending laboratory and that obtained by MALDI-TOF at the NRCCH were identified (33/2,360, 1.4%). For all these discrepancies, the identification obtained by WGS was consistent with that obtained by MALDI-TOF (data not shown). A total of 332 discrepancies (14%) between the resistance phenotypes obtained by the sending laboratory and the resistome were identified: 253 discrepancies for *C. jejuni* (76.2%), 76 for *C. coli* (22.9%), and 3 for *C. fetus* (0.9%). These included ampicillin (*n* = 127, 38.3%), ciprofloxacin (*n* = 76, 22.9%), tetracycline (*n* = 75, 22.6%), amoxicillin-clavulanic acid (*n* = 19, 5.7%), gentamicin (*n* = 17, 5.1%), and erythromycin (*n* = 18, 5.4%). Each discrepancy was resolved when additional AST for ampicillin, amoxicillin-clavulanic acid, ciprofloxacin, erythromycin, and gentamicin was performed at the NRCCH. Therefore, a predictive performance of 100% was obtained for these antibiotics. With respect to tetracycline, however, a small number of isolates (*n* = 11, 1%) were found to be susceptible despite carrying a *tet*(O) gene or any variant. In fact, as previously described, some *Campylobacter* isolates may not express the *tet* gene at the same level (33).

**TABLE 1:** Age and sex characteristics of the *Campylobacter* spp.-infected patients included in this study

	N	M	F	Sex ratio	Mean age (SD)
*C. jejuni*	1,959	1,137	822	1.4	34.9 (27.5)
*C. coli*	358	193	165	1.2	40.5 (27.7)
*C. fetus*	43	23	20	1.1	76.3 (13.3)
All species	2,360	1,353	1,007	1.3	36.5 (28)

**TABLE 2:** Isolation sites of the *Campylobacter* spp. sequenced in 2024

Origin	Total	*C. jejuni*	*C. coli*	*C. fetus*
Stools	2,265 (96%)	1,912 (97.6%)	350 (97.8%)	3 (7%)
Blood	89 (3.8%)	46 (2.3%)	7 (2%)	36 (83.7%)
Intestinal biopsy	2 (0.08%)	1 (0.05%)	1 (0.3%)	0
Articular liquid	4 (0.2%)	0	0	2 (4.5%)
Cerebrospinal fluid	2 (0.08%)	0	0	2 (4.5%)

### WGS detection of antibiotic resistance

Mutations in the quinolone-determining region (QRDR) of the *gyrA* gene associated with fluoroquinolone resistance were identified in 1,391 genomes (1,391/2,360, 58.9%): 1,142/1,959 *C. jejuni* (58.3%-R), 244/358 *C. coli* (68.2%-R), and 5/43 *C. fetus* (11.6%-R). T86I was the main mutation identified in *C. jejuni* and *C. coli*. The other mutations identified were rare (Table 3). Only one *C. jejuni* strain and four *C. coli* strains exhibited the double mutation T86I + D90N. D91Y and T87I mutations were identified only in *C. fetus*. T86V was detected only in one *C. coli sample*.

**TABLE 3:** Mutations in the quinolone-determining region (QRDR) of the *gyrA* gene identified in this study

QRDR mutation	*C. jejuni*	*C. coli*	*C. fetus*	All species
T86I	1,139	239	0	1,378 (99.1%)
T87I	0	0	3	3 (0.2%)
T86R	2	0	0	2 (0.1%)
T86V	0	1	0	1 (0.07%)
D91Y	0	0	2	2 (0.1%)
T86I + D90N	1	4	0	5 (0.4%)
	1,142/1,959 (58.3%)	244/358 (68.2%)	5/43 (11.6%)	1,391/2,360 (58.9%)

The main mechanism of erythromycin resistance identified in *C. jejuni* and *C. coli* was the presence of a mutation in the *23S rDNA* (93.6% of strains) (Table 4). No resistance mechanism was identified in *C. fetus*. Resistance was more frequent in *C. coli* than in *C. jejuni* (14.8% vs. 0.5%, respectively). The distribution of mutations was more diverse in *C. jejuni* than in *C. coli,* where the A2075G mutation was predominant (48 *C. coli* out of 53 isolates, 90.6%). Rare strains of *C. coli* possessed a chromosomal copy of the *erm*(N) gene (*n* = 2, with an erythromycin MIC of 32 mg/L and ≥256 mg/L) or the *erm*(B) gene (*n* = 1, with an erythromycin MIC of ≥256 mg/L). Two *C. coli* isolates carried an *erm*(A) gene within a resistance island containing the *catA9*, *tet*(O), *tet*(L), *ant* (9)-I, and *ant* (6)-I genes. The resistance island is closely related to that of *Enterococcus faecalis* pE508 and is linked to an increased erythromycin MIC (34). One isolate expressed only *erm*(A) (MIC of 16 mg/L), and one had *erm*(A) in combination with the *23S* A2075G mutation (MIC of 256 mg/L).

**TABLE 4:** Distribution of mechanisms associated with erythromycin resistance^*a*^

Resistance marker	*C. jejuni*	*C. coli*	*C. fetus*	All species
A2074C	1	1	0	2 (3.2%)
A2074T	4	0	0	4 (6.3%)
A2075G	5	48	0	53 (84.1%)
*erm(A*)	0	2	0	2 (3.2%)
*erm(B*)	0	1	0	1 (1.6%)
*erm(N*)	0	2	0	2 (3.2%)
	10/1,959 (0.5%)	53/358 (14.8%)	0/43	63/2,360 (2.7%)

^*a*^ *erm*: erythromycin methyltransferase gene.

A tetracycline resistance gene was identified in 1,021 of 2,360 strains (43.3%). *C. coli* was more resistant (84.6%) than *C. jejuni* (36.4%) and *C. fetus* (11.6%) (Table 5). The main gene found was *tet(O)* (chromosomal or plasmidic; data not shown) or its variants. *tet (44*) was identified only in *C. fetus*, whereas *tet(0-W-32-0*) and *tet(W*) were identified only in *C. coli*.

**TABLE 5:** Distribution of mechanisms associated with tetracycline resistance

Resistance marker	*C. jejuni*	*C. coli*	*C. fetus*	All species
*tet (44*)	0	0	3	3 (0.28%)
*tet(0*)	409	179	2	590 (57.41%)
*tet(0-32-0*)	299	108	0	407 (39.57%)
*tet(0-M-0*)	5	10	0	15 (1.51%)
*tet(0-W-32-0*)	0	1	0	1 (0.09%)
*tet (W*)	0	5	0	5 (0.57%)
	713/1,959 (36.4%)	303/358 (84.6%)	5/43 (11.6%)	1,021/2,360 (43.3%)

Fourteen strains carried a gentamicin resistance gene in their genome: 3 *C. jejuni* (0.1%) and 11 *C. coli* (3.1%). The *aph(2’’)-Ii1* gene was found in eight genomes, including six *C. coli*, and the *aph(2’’)-IIIa* gene was found only in *C. coli* (four strains). Finally, *aph(2’’)If* was detected in two strains (one *C. jejuni* strain and one *C. coli* strain).

Kanamycin resistance was carried solely by the *aph(3’)-IIIa* gene in 25 *C. jejuni* strains (1.3%), 23 *C. coli* strains (6.4%), and 2 *C. fetus* strains (4.7%). Spectinomycin resistance was carried either by the *ant (9)-Ic-aad9 gene* (4 *C. jejuni* and 11 *C. coli*) or by the *spw* gene in only 3 *C. coli* strains. One *C. coli* strain carried both one copy of the *ant (9)-Ic-aad9* gene and one copy of the *spw* gene. Spectinomycin resistance was detected in 4 *C. jejuni* (0.2%) and 15 *C. coli* (4.2%) strains.

Genes associated with streptomycin resistance were detected in 214 genomes (Table 6). *C. coli* was more resistant (35.8%) compared with *C. jejuni* (4.1%) and *C. fetus* (11.6%). The main enzyme identified in *C. jejuni* and *C. coli* was *ant (6)-If-aadE* (*n* = 124, 58.18%). An *ant (6)-Ig* was found only in 54 *C. coli* strains. Four *C. coli* strains carried two genes associated with streptomycin resistance: *ant (6)-If-aadE + ant (6)-Ig* (*n* = 2) or *ant (6)-If-aadE + ant (6)-Ia* (*n* = 2). A gene encoding *ant (6)-Ib* was identified in only three *C. fetus* strains.

**TABLE 6:** Distribution of mechanisms associated with streptomycin resistance

Resistance marker	*C. jejuni*	*C. coli*	*C. fetus*	All species
*ant (6)-Ib*	0	0	3	3 (1.37%)
*ant (6)-If-aadE*	63	61	0	124 (58.18%)
*ant (6)-Ig*	0	54	0	54 (25.45%)
*ant (6)-Ia*	18	9	2	29 (13.18%)
*ant (6)-If-aadE + ant (6)-Ig*	0	2	0	2
*ant (6)-If-aadE + ant (6)-Ia*	0	2	0	2
	81/1,959 (4.1%)	128/358 (35.8%)	5/43 (11.6%)	214/2,360 (9.1%)

Resistance to group A penicillins was absent from all the *C. fetus* strains sequenced in the present study. No beta-lactamase gene was detected in 347 genomes (14.7%): 8.73% for *C. jejuni* and 37.15% for *C. coli*. A great diversity of beta-lactamase genes was identified among the 2,013 *bla_OXA_*-positive isolates (Table 7), with 36 variants in total. *bla_OXA_*193 was the most frequent gene (*n* = 1,270, 63.1%), followed by *bla_OXA_*61 (*n* = 114, 5.7%) and *bla_OXA_*461 (*n* = 111, 5.5%). A total of 694 isolates (29.4%) were found to be resistant *in vitro* to group A penicillins but remained susceptible to amoxicillin-clavulanic acid. An exception was observed in four *C. coli* isolates that harbored an A51G deletion in the *bla_OXA_* promoter and exhibited an amoxicillin-clavulanic acid MIC ≥256 mg/L (21). As previously described, the expression of *bla_OXA_* genes is dependent on the sequence of the promoter (35). The G57T mutation in the promoter was detected in 586 of the 2,013 beta-lactamase-positive strains. A total of 90 strains (all *C. jejuni*) harbored an undescribed promoter sequence (named PROM2024), which corresponded to the year of the first identification of this new promoter allowing the expression of the *bla_OXA_* gene by itself. Finally, 20 *C. jejuni* isolates harbored a G58T mutation, and two *C. jejuni* isolates had a 6-nucleotide deletion in their promoter region (del6NT, Table 7). Additional MICs were determined for a selection of WT, G57T, PROM2024, and both del6NT isolates. The results are shown in Table S2. With the exception of four PROM2024 isolates with low MICs (possibly due to a mutation within the TATA Pribnow box), all the tested non-WT isolates were resistant to group A penicillins.

**TABLE 7:** Distribution of resistance mechanisms to group A penicillin^*b*^

*blaOXA* (promoter)	*C. jejuni*		*C. coli*		*C. fetus*		All species	
	n	%	n	%	n	%	n	%
WT *bla_OXA_^a^*	1188	60.64%	127	35.47%			1315	55.72%
No *bla_OXA_* gene^*a*^	171	8.73%	133	37.15%	43	100%	347	14.70%
*bla_OXA_*193-G57T	332	16.95%	62	17.32%			394	16.69%
*bla_OXA_*61-G57T	91	4.65%	2	0.56%			93	3.94%
*bla_OXA_*184-PROM2024	71	3.62%					71	3.01%
*bla_OXA_*489-G57T	6	0.31%	21	5.87%			27	1.14%
*bla_OXA_*461-G57T	22	1.12%	1	0.28%			23	0.97%
*bla_OXA_*193-G58T	20	1.02%					20	0.85%
*bla_OXA_*451-G57T	12	0.61%					12	0.51%
*bla_OXA_*460-G57T	10	0.51%					10	0.42%
*bla_OXA_*595-G57T	8	0.41%	2	0.56%			10	0.42%
*bla_OXA_*633-PROM2024	9	0.46%					9	0.38%
*bla_OXA_*594-G57T	1	0.05%	8	2.23%			9	0.38%
*bla_OXA_*625-PROM2024^*a*^	4	0.20%					4	0.17%
*bla_OXA_*465-PROM2024	3	0.15%					3	0.13%
*bla_OXA_*584-G57T	2	0.10%	1	0.28%			3	0.13%
*bla_OXA_*452-G57T	1	0.05%	1	0.28%			2	0.08%
*bla_OXA_*193-Del6NT	2	0.10%					2	0.08%
*bla_OXA_*466-PROM2024	2	0.10%					2	0.08%
*bla_OXA_*577-G57T	1	0.05%					1	0.04%
*bla_OXA_*592-G57T	1	0.05%					1	0.04%
*bla_OXA_*591-G57T	1	0.05%					1	0.04%
*bla_OXA_*630-PROM2024	1	0.05%					1	0.04%

^*a*^ Susceptible isolates.

^*b*^ *bla_OXA_*: beta-lactamase; PROM: promoter; NT: nucleotides; WT: wild-type.

In this study, the most frequently encountered antibiotic resistance profiles for the *Campylobacter* spp. sequenced are presented in Table S3. Resistance to fluoroquinolones only was detected in 402 strains (17%), followed by a dual resistance to fluoroquinolone and tetracycline in 360 strains (15.2%). Finally, 266 strains (9.6%) exhibited a resistance profile that is typically found in fewer than 10% of strains.

### Source attribution for *C. jejuni* and *C. coli*

In terms of all the sources combined, *C. jejuni* strains were predominantly attributed to the poultry reservoir (64.2%), followed by the ruminant reservoir (29.9%), the environment (2.9%), or an unidentifiable reservoir (3.1%). This distribution was stable between strains isolated from stool and those from blood cultures (Table 8). For *C. coli*, a source was attributed to all the strains, and poultry was also the main reservoir (90.5%), far ahead of pigs (6.2%) and ruminants (0.3%) (Table 9). The small number of strains isolated from blood culture bottles did not allow for a comparative analysis of potential sources of contamination. There is currently no source attribution marker described in the literature for *C. fetus*.

**TABLE 8:** Attribution of *C. jejuni* contamination sources

	Poultry	Ruminant	Environment	ND^*a*^
All *C. jejuni* (*n* = 1,959)	1,257 (64.2%)	585 (29.9%)	56 (2.9%)	61 (3.1%)
Stools (*n* = 1,912)	1,277 (66.8%)	573 (30%)	52 (2.7%)	60 (3.1%)
Blood (*n* = 46)	29 (63%)	12 (26.1%)	4 (8.7%)	1 (2.2%)
Intestinal biopsy (*n* = 1)	1	0	0	0

^*a*^ ND, not determined.

**TABLE 9:** Attribution of sources of *C. coli* contamination

	Poultry	Ruminant	Pig	ND^*a*^
All *C. coli* (*n* = 368)	333 (90.5%)	1 (0.3%)	23 (6.2%)	0
Stools (*n* = 350)	328 (97.7%)	1 (0.3%)	20 (5.7%)	0
Blood (*n* = 7)	5 (71.4%)	0	2 (28.6%)	0
Intestinal biopsy (*n* = 1)	0	1	0	0

^*a*^ ND, not determined.

### MLST and cgMLST analyses

Globally, the *C. jejuni* strains were divided into 16 CCs of at least 20 representatives, with CC-21 accounting for 33.18% of the strains (*n* = 650), as commonly reported by others (36, 37). CC-21 is predominant in both the poultry and the ruminant reservoir, representing 371 (29.5% of the poultry group) and 262 (44.8% of the ruminant group) isolates, respectively. CC-443 and CC-353 (with 121 and 109 isolates, respectively) are, however, exclusive to the poultry reservoir, as they are not found among ruminant-associated isolates. Greater diversity was found for sequence types (STs), with a heterogeneous distribution observed among 24 STs, each representing less than 8% of the strains. The first was ST-50, which represented only 7.6% of the strains (Table S4). For *C. coli*, however, the sequenced strains belonged primarily to CC-828, which represented 97.4% of the strains. The CC-828 isolates were predominant in each predicted source of contamination, namely, poultry, ruminants, and pigs. The strains were divided into only five major STs, the most frequent of which was ST-8195 (13.4%) (Table S5). No CCs or STs were identified for *C. fetus,* as this species is not included in the PubMLST database.

First, core-genome MLST (cgMLST) was run on the *C. jejuni* and *C. coli* genomes with a cutoff of 25 allelic variations (i.e., a cluster of strains forms when two strains have fewer than 25 different core genome genes) (data not shown). No major clusters were found for *C. jejuni*. However, for *C. coli*, a cluster comprising the ST-8195, ST-1628, and ST-10042 isolates was observed (these isolates represented 12.5% of the *C. coli* in the present study). All of the strains in this cluster had poultry as a potential source of contamination and were resistant to ciprofloxacin, tetracycline, and streptomycin. They were also distributed throughout the country.

By lowering the clustering threshold to four allelic variations, this cluster was no longer detected. Thus, it did not correspond to the spread of a clone epidemic. Specifically, when this threshold was used, the largest *C. jejuni* cluster identified in 2024 was composed of 58 isolates, representing 3% of the NRCCH *C. jejuni* strains, and the largest *C. coli* cluster was composed of 18 isolates, representing 5% of the NRCCH *C. coli* strains, as shown in Fig. S2. Moreover, the detected clusters were composed mainly of singletons (i.e., clusters composed of only one isolate): 1,183 for *C. jejuni* (60% of the total data set for that species) and 262 for *C. coli* (73% of the total data set for that species).

## DISCUSSION

The results presented in our study provide an overview of the main mechanisms of antibiotic resistance currently found in France for the three main *Campylobacter* pathogenic species. Using this strategy, we identified a variety of resistance mechanisms and the low-level circulation of genetically related strains of *C. coli* in France during 2024, which did not constitute a large-scale epidemic spread of a clone.

*C. jejuni* was the predominant species in our study, which is consistent with the epidemiology of *Campylobacter* spp. infections in France and other industrialized countries (36, 38). The role of *C. fetus* was almost exclusively limited to strains isolated from blood culture bottles. This phenomenon is probably an epidemiological bias linked to the use in biological laboratories of so-called syndromic PCR detection in stool samples for *Campylobacter*, which does not allow *C. fetus* to be detected (6, 39). However, on the basis of all the data, 2024 *C. fetus* accounted for 0.14% of all *Campylobacter* isolated from stool samples (0.7% when considering all the sample types) and was sent to the NRCCH for analysis (https://www.cnrch.fr/wp-content/uploads/Rapport-2025-CNRCH-activite-2024_V2.pdf).

Poultry is the main source of *C. jejuni* and *C. coli* infection (18–20). In France, ruminants account for a significant proportion of *C. jejuni* infections, whereas pigs account for a more limited proportion of *C. coli* infections. These data are consistent with our previous analyses and differ from data from other countries (40). In the U.S., pigs account for a relatively large proportion of *C. coli* infections (19). Since 2023, half of the poultry consumed in France has been imported (notably from Poland, Ukraine, and Belgium); hence, the emergence of clusters is an important factor to monitor (https://www.agreste.agriculture.gouv.fr/agreste-web/download/publication/publie/SynAvi24426/consyn426202407-Aviculture.pdf). However, the lack of a policy of mass sequencing in these countries for strains potentially present in reservoirs prevents any comparison at the European level from a One Health perspective (https://storymaps.arcgis.com/stories/37987745de6f47029e14cb57d61fe923). In our study, the main CCs identified in *C. jejuni* human isolates were attributed to both chicken and ruminant reservoirs. These results underscore the significance of these sources in terms of human contamination in our country. However, the CC and ST types of MLST can vary by country. For instance, a recent Italian study revealed different proportions of CC and ST types (41). ST21, the strain of *C. jejuni* considered to be the most virulent, represented only 5.31% of the *C. jejuni* isolates sequenced in our study. Thus, the epidemiology of campylobacteriosis is likely influenced by dietary habits and food origin.

Resistance to macrolides has remained low in France for the three most common species (16). This is not the case in some European countries, with the highest proportions of resistance in *C. coli* observed in Greece (38.5), Portugal (26.9%), and Spain (19.3%) (https://www.ecdc.europa.eu/sites/default/files/documents/CAMP_AER_2022_final.pdf). However, the percentage of *C. coli* detected as non-wild type (NWT) among the 358 sequenced isolates in France was greater in 2024 than the percentage of all erythromycin-resistant *C. coli* isolates received at the NRCCH based on AST data: 53/358 for WGS (15%) versus 61/1,062 for AST (5.7%). This difference may be because some laboratories send only a small proportion of all the *Campylobacter* strains they cultivate, particularly erythromycin-resistant strains, resulting in an epidemiological bias. In a previous study, we reported an increase in the percentage of *erm*-positive strains, particularly *C. coli* (42). This increase was not confirmed in 2024, but *erm*-positive strains remain present in both species (Table 4). Fortunately, no *erm(B*) genes were carried by a plasmid, which is reassuring in terms of the absence of risk of an epidemic outbreak in this case. However, we are continuing to monitor the emergence of *erm*-positive strains in France using WGS. Interestingly, no mutations, insertions, or deletions in the ribosomal proteins L4 and L22, which are encoded by the *rplD* and *rplV* genes, respectively, were detected among the erythromycin-resistant isolates, as reported by others (43). As previously published, erythromycin-resistant strains have a multidrug resistance profile (16), indicating strong historical selection pressure in the reservoirs studied.

Resistance to fluoroquinolones is high in France and is present in all reservoirs identified for *C. jejuni* and *C. coli* (data not shown). The main mutation found is T86I, which has already been described by other authors (44, 45), indicating that in *Campylobacter,* a single point mutation in *gyrA* can lead to a high level of resistance to fluoroquinolones, whereas a high level of resistance to fluoroquinolones usually requires the accumulation of successive point mutations in other bacterial genera (46). This is also true for *C. fetus*. In our study, no *qnr* genes were detected in *Campylobacter* (47).

Resistance to tetracycline is highly diverse, with a multitude of *tet* close to each other. The majority of *tetO* and the mosaic gene *tet(0-32-0)* (48) are carried either by the chromosome or plasmids. As described previously, resistance to tetracycline is very common, not only in *C. coli* but also in *C. jejuni* (49).

Unlike in Asian countries (50), resistance to gentamicin remains very low in France and in other EU countries (except Spain and Greece, which reported higher resistance rates in *C. jejuni* (3.6%) and *C. coli* (23.1%) in 2022, respectively), but the presence of genes carried by plasmids in certain strains should be monitored. Resistance to other aminoglycosides, however, is more common, particularly to streptomycin, spectinomycin, and kanamycin. These genes are often present within genomic resistance islands (51). However, gentamicin remains the preferred aminoglycoside in combination with a group A penicillin for the treatment of invasive infections (52); thus, resistance to other aminoglycosides has no effect on human clinical practice.

Resistance to group A penicillin is linked to the presence of a gene encoding a chromosomal beta-lactamase, the expression of which is determined by the promoter sequence. In our 2024 data, we observed a wide variety of *bla_oxa_* genes that differed from each other by a few SNPs. As described by Zeng X et al. (35), G57T is the most common mutation, and its presence correlates perfectly with *in vitro* resistance. In the present study, we describe a novel promoter that has not yet been described in the literature (PROM2024). We have integrated it into our analysis pipeline, although further experiments are needed in the future to characterize it properly. Despite the sequence of the *bla_oxa_* promoter, clavulanic acid retains its anti-beta-lactamase activity. Only one strain of *C. coli* was resistant to amoxicillin-clavulanic acid because of the presence of a deletion in A51G in the *bla_oxa_*61 promoter, as previously published by our team (21). These results confirm the value of this combination in the treatment of *Campylobacter* infections, particularly in bacteremia, as proposed by Tinevez et al. (52). The role of amoxicillin-clavulanate remains debated, and some guidelines favor the use of carbapenems as first-line therapy because of the broader coverage and lower risk of failure when the infection source is uncertain or uncontrolled. In our opinion, general antibiotic use policies should restrict carbapenem use to severe infections and bacteria that are resistant to other antibiotics. According to 33 years of monitoring by the NRCCH, resistance to amoxicillin-clavulanate is nonexistent in *C. jejuni* and rare in *C. coli* in France (53). Therefore, amoxicillin-clavulanate remains the first-line therapeutic option for any invasive *Campylobacter* spp. infection in the absence of allergy. This phenomenon was also demonstrated in a recent multicenter French study on the epidemiology and treatment of *Campylobacter* spp. bacteremias (52).

As described in the UK in 2021 by Swift et al. (36), the routine use of cgMLST analysis in the context of outbreak investigations allowed us to confirm that no distinctive and alarming *Campylobacter* clone has been spreading across the country since 2024. A wide variety of strains have been observed, specifically for *C. jejuni* isolates (Fig. S2), which may indicate that consumers are exposed to multiple sources of contamination.

For *C. coli*, it appears that certain genetically related strains circulated during 2024. Since January 2025, however, this cluster has declined (data not shown). Our results contrast with those in other countries, where specific clones can regularly be observed (54). Although clinical *Campylobacter* spp. isolates related to reported outbreaks in neighboring countries have been detected in France, their prevalence remains limited, which may be due to the large-scale sequencing strategy employed. The strict monitoring of *Campylobacter* spp. clones is ongoing and will be further improved in the coming years through collaborative studies with the French Public Health Agency (SPF) and the French Agency for Food Health and Safety (ANSES).

Of course, our study has several limitations because it is based on only some of the strains we received in 2024. Nevertheless, we believe that the data presented shed new light on *Campylobacter* spp. infections, particularly in France. In the coming years, we will monitor the data on antibiotic resistance and source attribution, which may serve as a basis for comparative studies for other laboratories wishing to implement an NGS approach in routine studies of *Campylobacter* spp. infections.

## Data Availability

Genomes are available to download as assembly fasta files in the ABRomics database https://analysis.abromics.fr/projects/access-eyJQMUxSSkhXWjJGIjoyMjV9:1vU4kt:clsDcWlioKH4YeNu8JWWRiUEQU363nO8rn7zXu2YW-A or by using the ENA accession numbers indicated in Table S1 (project number PRJEB105069).

## SUPPLEMENTAL MATERIAL

Fig. S1 aac.00193-26-s0001.docx Geographical origin of the *Campylobacter* spp. clinical isolates sequenced in 2024.

Fig. S2 aac.00193-26-s0002.docx Core-genome MLST trees for *C. jejuni* (left) and *C. coli* (right) designed from the hierarchical clustering algorithm of ReporTree and GrapeTree rendering tools.

Table S1 aac.00193-26-s0003.xlsx Complete data table of the studied isolates.

Table S2 aac.00193-26-s0004.xlsx Minimum inhibitory concentrations to group A penicillins performed on a selection of WT blaOXA promoters, G57T mutated blaOXA promoters, Del6NT blaOXA promoters, and blaOXA PROM2024 isolates.

Table S3 aac.00193-26-s0005.docx Main antibiotic resistance markers profiles in sequenced *Campylobacter* spp. isolates.

Table S4 aac.00193-26-s0006.docx Distribution of clonal complexes and sequence types for *C. jejuni* strains.

Table S5 aac.00193-26-s0007.docx Distribution of clonal complexes and sequence types for *C. coli* strains.

ASM does not own the copyrights to Supplemental Material that may be linked to, or accessed through, an article. The authors have granted ASM a non-exclusive, world-wide license to publish the Supplemental Material files. Please contact the corresponding author directly for reuse.
