# Supplementary material for: Contributions of whole-genome sequencing to the epidemiological monitoring of Campylobacter spp. in France
Source: Antimicrob Agents Chemother. 2026 May 29;70(7):e00193-26. doi: 10.1128/aac.00193-26 (PMC13321834; doi:10.1128/aac.00193-26)
Supplement: Fig. S1 — Geographical origin of the Campylobacter spp. clinical isolates sequenced in 2024. [file aac.00193-26-s0001.docx]

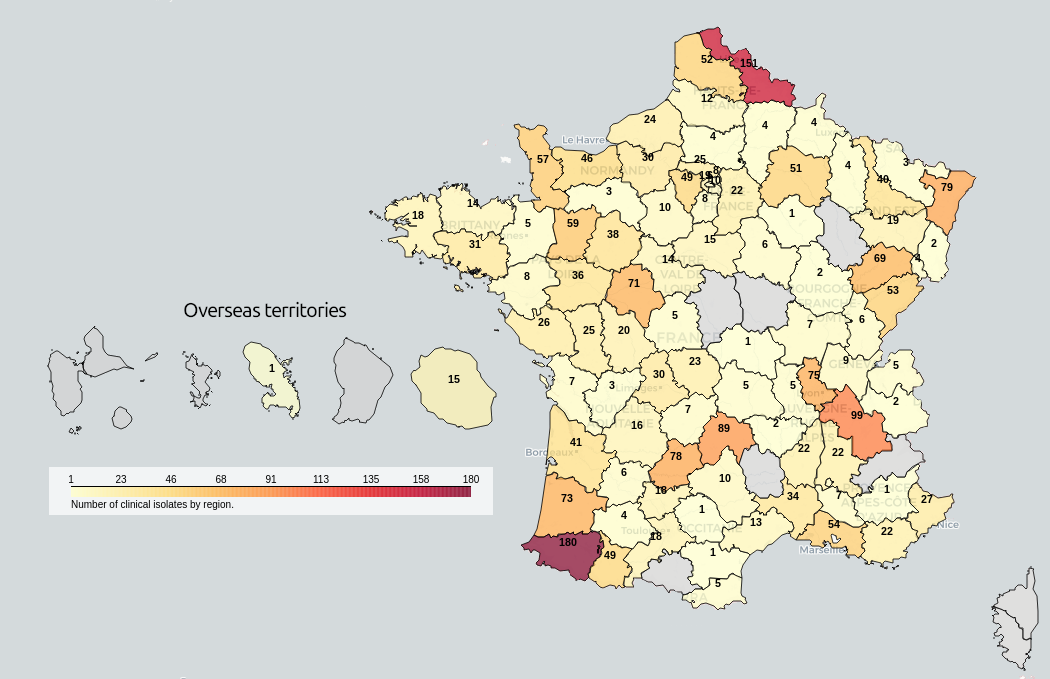


**Supp Figure 1.** Geographical origin of the *Campylobacter* spp. clinical isolates sequenced in 2024. A total of 2,360 clinical isolates of *C. jejuni* (n=1,959), *C. coli* (n=358) or *C. fetus* (n=43) are displayed here. Five isolates were also sampled from French overseas communities (4 from French Polynesia and 1 from New Caledonia).
