# Supplementary material for: Contributions of whole-genome sequencing to the epidemiological monitoring of Campylobacter spp. in France
Source: Antimicrob Agents Chemother. 2026 May 29;70(7):e00193-26. doi: 10.1128/aac.00193-26 (PMC13321834; doi:10.1128/aac.00193-26)
Supplement: Table S3 — Main antibiotic resistance markers profiles in sequenced Campylobacter spp. isolates. [file aac.00193-26-s0005.docx]

**Supplemental Table 3.** Main antibiotic resistance markers profiles in sequenced *Campylobacter* spp. isolates.

| **Resistance marker** | **Mutations or resistance genes** | **All species** | |
| --- | --- | --- | --- |
| None | - | 638 | 27% |
| Q | GyrA-T86I | 402 | 17% |
| QT | GyrA-T86I;*tet(O)* | 241 | 10.2% |
| QT | GyrA-T86I;*tet(O-32-O)* | 119 | 5% |
| QTA | GyrA-T86I; *bla_OXA_*193 (G57T);*tet(O-32-O)* | 97 | 4.1% |
| QA | GyrA-T86I; *bla_OXA_*193 (G57T) | 88 | 3.7% |
| T | *tet(O)* | 77 | 3.3% |
| QTA | GyrA-T86I; *bla_OXA_*193 (G57T);*tet(O)* | 66 | 2.8% |
| QTStr | GyrA-T86I;*tet(O-32-O) ;ant(6)-If-aadE*; | 66 | 2.8% |
| A | *bla_OXA_*61 (G57T) | 59 | 2.5% |
| A | *bla_OXA_*193 (G57T) | 41 | 1.7% |
| QTAStr | GyrA-T86I; *bla_OXA_*193 (G57T);*tet(O-32-O); ant(6)-If-aadE* | 38 | 1.6% |
| T | *tet(O-32-O)* | 29 | 1.2% |
| QTA | GyrA-T86I; *bla_OXA_*184 (PROM2024);*tet(O)* | 24 | 1% |
| QA | GyrA-T86I; *bla_OXA_*184 (PROM2024) | 20 | 0.8% |
| QTA | GyrA-T86I; *bla_OXA_*193 (G58T);*tet(O)* | 19 | 0.8% |
| AT | *bla_OXA_*193 (G57T);*tet(O)* | 19 | 0.8% |
| QTA | GyrA-T86I; *bla_OXA_*461 (G57T);*tet(O)* | 17 | 0.7% |
| A | *bla_OXA_*184 (PROM2024) | 16 | 0.7% |
| AT | *bla_OXA_*193 (G57T);*tet(O)* | 14 | 0.59% |
| A | *bla_OXA_*193 (G57T) | 12 | 0.5% |
| QTAKStr | GyrA-T86I; *bla_OXA_*193 (G57T);*tet(O-32-O);aph(3')-IIIa*;*sat-4*; | 11 | 0.5% |
| QTStr | GyrA-T86I;*tet(O);ant(6)-Ig* | 11 | 0.5% |
| QTA | GyrA-T86I; *bla_OXA_*460 (G57T);*tet(O)* | 10 | 0.4% |
| - | Other combinations (less than 10 strains for each) | 226 | 9.6% |

Q: quinolones; T: tetracycline; A: groupA-penicillins; K: kanamycin; Str: streptomycin; *blaOXA*: betalactamase.
