# Supplementary material for: Contributions of whole-genome sequencing to the epidemiological monitoring of Campylobacter spp. in France
Source: Antimicrob Agents Chemother. 2026 May 29;70(7):e00193-26. doi: 10.1128/aac.00193-26 (PMC13321834; doi:10.1128/aac.00193-26)
Supplement: Table S4 — Distribution of clonal complexes and sequence types for C. jejuni strains. [file aac.00193-26-s0006.docx]

**Supplemental Table 4.** Distribution of clonal complexes and sequence types for *C. jejuni* strains.

| **Clonal complexes** | **No.** | **%** |
| --- | --- | --- |
| CC-21 | 650 | 33.18% |
| CC-nd | 145 | 7.4% |
| CC-48 | 130 | 6.64% |
| CC-443 | 121 | 6.18% |
| CC-353 | 111 | 5.67% |
| CC-206 | 106 | 5.41% |
| CC-45 | 81 | 4.13% |
| CC-49 | 70 | 3.57% |
| CC-52 | 66 | 3.37% |
| CC-42 | 65 | 3.32% |
| CC-22 | 63 | 3.22% |
| CC-354 | 54 | 2.76% |
| CC-257 | 51 | 2.6% |
| CC-658 | 45 | 2.3% |
| CC-464 | 36 | 1.84% |
| CC-283 | 34 | 1.74% |
| CC-607 | 30 | 1.53% |
| CC-460 | 22 | 1.12% |
| CC-61 | 21 | 1.07% |
| **Others** | **58** | **2.96%** |
|  |  |  |
| **Sequence types** | **No.** | **%** |
| ST-50 | 149 | 7.61% |
| ST-19 | 147 | 7.5% |
| ST-51 | 110 | 5.62% |
| ST-21 | 104 | 5.31% |
| ST-48 | 68 | 3.47% |
| ST-49 | 66 | 3.37% |
| ST-122 | 58 | 2.96% |
| ST-52 | 58 | 2.96% |
| ST-10298 | 56 | 2.86% |
| ST-22 | 54 | 2.76% |
| ST-354 | 46 | 2.35% |
| ST-45 | 44 | 2.25% |
| ST-464 | 36 | 1.84% |
| ST-475 | 36 | 1.84% |
| ST-6532 | 34 | 1.74% |
| ST-10846 | 33 | 1.68% |
| ST-257 | 26 | 1.33% |
| ST-14316 | 25 | 1.28% |
| ST-441 | 25 | 1.28% |
| ST-6089 | 25 | 1.28% |
| ST-6175 | 25 | 1.28% |
| ST-267 | 22 | 1.12% |
| ST-42 | 22 | 1.12% |
| ST-1044 | 20 | 1.02% |
| **Others** | **670** | **34.2%** |

Others: less than 20 strains for each CC or ST. CC-nd: CC not determined.
