# Supplementary material for: Contributions of whole-genome sequencing to the epidemiological monitoring of Campylobacter spp. in France
Source: Antimicrob Agents Chemother. 2026 May 29;70(7):e00193-26. doi: 10.1128/aac.00193-26 (PMC13321834; doi:10.1128/aac.00193-26)
Supplement: Table S5 — Distribution of clonal complexes and sequence types for C. coli strains. [file aac.00193-26-s0007.docx]

**Supplemental Table 5.** Distribution of clonal complexes and sequence types for *C. coli* strains.

| **Clonal complexes** | **No.** | **%** |
| --- | --- | --- |
| CC-828 | 345 | 96.37% |
| **Others** | **13** | **3.63%** |
|  |  |  |
| **Sequence types** | **No.** | **%** |
| ST-8195 | 48 | 13.41% |
| ST-827 | 40 | 11.17% |
| ST-825 | 34 | 9.5% |
| ST-832 | 24 | 6.7% |
| ST-1770 | 21 | 5.87% |
| **Others** | **191** | **53.35%** |

Others: less than 20 strains for each CC or ST.
